# Comparison of biochemical and hematologic values obtained via jugular venipuncture and peripheral intravenous catheters in dogs

**DOI:** 10.1111/jvim.16518

**Published:** 2022-08-27

**Authors:** Aria L. Guarino, Andrew J. Specht, Sarah S. K. Beatty, Allison L. O'Kell

**Affiliations:** ^1^ University of Florida Department of Small Animal Clinical Sciences College of Veterinary Medicine Gainesville Florida USA; ^2^ Blue Pearl Veterinary Partners Rockville Maryland USA; ^3^ University of Florida Department of Comparative Diagnostic and Population Medicine College of Veterinary Medicine Gainesville Florida USA; ^4^ Antech Diagnostics Fountain Valley California USA

**Keywords:** Bland‐Altman analysis, complete blood count, Passing‐Bablok regression, serum chemistry

## Abstract

**Background:**

Sampling from a peripheral intravenous catheter (PIVC) might be a more efficient and less traumatic collection of blood for serum biochemistry (SB) or CBC than direct venipuncture (DV). Agreement between results of samples obtained by these methods has not been evaluated in dogs.

**Objectives:**

The primary objectives were to determine whether sampling from PIVC could be used in place of DV for dogs. We hypothesized DV and PIVC samples would have clinically equivalent SB and CBC results.

**Animals:**

Sixty‐one client‐owned dogs were included in each study arm.

**Methods:**

This was a partially randomized method‐comparison study. Paired DV and PIVC samples obtained within 1 to 2 minutes after, or approximately 24 hours after, placement of a PIVC in a cephalic vein were evaluated for agreement and bias using percentage difference plots (with a priori application of consensus total allowable error), Bland‐Altman analysis, Passing‐Bablok regression analysis, Wilcoxon signed rank test, and McNemar's test.

**Results:**

There was statistically and clinically acceptable agreement and no bias between sampling methods for the majority of results. Analytes with the most frequent disagreement were aspartate aminotransferase, total bilirubin, potassium, bicarbonate, and leukocyte differential counts, as well as red blood cell count, hemoglobin, hematocrit, and packed cell volume in the hospitalized PIVC sampling group. Few observed differences would change clinical decision making.

**Conclusions and Clinical Importance:**

PIVC sampling can provide generally acceptable SB and CBC results for most dogs, but clinicians should be aware of a few values for which disparate results might occasionally be obtained.

**Abbreviations**
ALB: albumin
ALP: alkaline phosphatase
ALT: alanine aminotransferase
AST: aspartate aminotransferase
AU480: Beckman Coulter AU480 biochemistry analyzer
BAND: band neutrophil count
BASO: basophil count
BUN: blood urea nitrogen
CA: total calcium
CHOL: cholesterol
CI: confidence interval
CL: chloride
CREA: creatinine
cTEa: consensus total allowable error
CV: coefficient of variation
DIFF: leukocyte differential counts
DV: direct venipuncture
EOS: eosinophil count
GLU: glucose
HB: hemoglobin
HCO3: bicarbonate
HCT: hematocrit
HDW: hemoglobin distribution width
hPIVC: peripheral intravenous catheter in a hospitalized patient
HPP: heat precipitated proteins
fPIVC: freshly placed peripheral intravenous catheter
K: potassium
LoA: limits of agreement
LIH: lipemia, icterus, hemolysis
LYMPH: lymphocyte count
MCHC: mean corpuscular hemoglobin concentration
MCV: mean corpuscular volume
MG: magnesium
MPV: mean platelet volume
NA: sodium
NEUT: neutrophil count
nRBC: nucleated red blood cell count
PHOS: phosphorus
PIVC: peripheral intravenous catheter
PLT: total platelet count
QGI: quality goal index
RBC: red blood cell count
RI: reference interval
RDW: red cell distribution width
SB: serum biochemistry
TBIL: total bilirubin
TE_obs_
: observed total error
TP: total protein
WBC: leukocyte count

## 1 INTRODUCTION

For many dogs presented for medical evaluation, it could be necessary to collect a blood sample to perform serum biochemistry (SB) and CBC. It is possible to obtain blood samples directly from a peripheral intravenous catheter (PIVC), either freshly placed (fPIVC) or after a period of hospitalization (hPIVC)^1^, ^2^ but whether these samples provide reliable SB and CBC results is unclear.

In humans, collection of blood samples from PIVC rather than direct venipuncture (DV) is recommended for patients who are pediatric, have difficult vascular access, have coagulopathies, or need repeat testing.^3^, ^4^ Using PIVC sampling limits patient discomfort, decreases needle injuries, and decreases ecchymoses compared to DV.^5^, ^6^ Human studies evaluating SB and CBC found no clinically important or statistically significant differences between collection methods for most or all analytes,^4^, ^7^, ^8^, ^9^, ^10^, ^11^, ^12^ though some studies found clinically important or statistically significant differences for bicarbonate (HCO_3_),^8^, ^13^ potassium (K),^10^, ^12^, ^13^ or glucose (GLU).^6^, ^13^

Clinical confidence in PIVC blood sampling for SB and CBC might reduce the need for DV in dogs. DV commonly requires physical restraint of the dog and utilizes 2 or more people, whereas collecting blood from a PIVC may require minimal restraint and a single individual. In dogs with cardiovascular compromise, respiratory distress, coagulopathies, small size, or fractious behavior, DV could be difficult, time‐consuming, or dangerous for the dog and staff. Obtaining blood from PIVC rather than DV could help improve staff efficiency as well as the dog's safety and comfort.^5^

The primary objectives of this study were to determine whether sampling from fPIVC or hPIVC approximately 1 day after placement can reliably be used for SB and CBC testing. The hypothesis was that SB and CBC results from fPIVC and hPIVC would be clinically equivalent to those from DV.

## 2 MATERIALS AND METHODS

### 2.1 Study design and groups

This was a 2‐arm prospective clinical study involving client‐owned dogs. The first arm was randomized and involved comparison of SB and CBC results from samples collected by DV and from fPIVC. The second arm was not randomized and involved comparison of SB and CBC results between DV and hPIVC 24 (±4) hours after placement. A dog could participate in both arms of the study if eligible.

Dogs were eligible for arm 1 if presented for clinical illness or procedures requiring a placement of a PIVC. Other inclusion criteria included: (a) blood sample collection during laboratory operating hours (to avoid delayed processing) and (b) a minimum body weight of 8 kg (because of volume of blood collected). Exclusion criteria included: (a) evidence of acute blood loss, (b) PCV < 20%, (c) evidence of vasculitis or coagulopathy such as petechiae, ecchymosis, subcutaneous edema, prolonged prothrombin or activated partial thromboplastin times, prolonged activated clotting time, thrombocytopenia of less than 70,000 platelets per microliter, or von Willebrand factor deficiency, or (d) inability to successfully perform jugular DV or place a cephalic vein PIVC.

Dogs were eligible for arm 2 if hospitalized with a 20‐gauge, 1.25″ cephalic vein PIVC in place for 24 ± 4 hours. Other inclusion and exclusion criteria were the same for arm 1, with additional criteria that (a) no other PIVC had been placed in that vein, (b) patency was documented (IV medication administration or saline flush every 4‐6 hours) from placement until sample collection, and (c) adequate sample volume could be collected.

The study was approved by the Institutional Animal Care and Use Committee and Veterinary Hospital Research Review Committee. Clients provided informed consent before their dog's participation.

### 2.2 Procedures

Direct venipuncture, PIVC placement, and PIVC blood collection was performed by one author (A. L. Guarino) or a licensed veterinary technician, and all procedures were witnessed by one of the authors. For both arms, DV and PIVC blood collection for paired samples were performed contemporaneously. In arm 1, the order of DV and fPIVC placement/sampling was randomized using a random number generator. Order of sampling was not randomized in arm 2 to prevent unnecessary DV for dogs in which the hPIVC did not provide an adequate blood sample.

For DV, a 6‐mL syringe with a 20‐gauge needle was used to collect 4‐6 mL of blood from a jugular vein with a “clean stick,” defined as no redirections of the needle once inserted into the vein and blood observed in the needle hub. Redirections through the skin were allowed. After successful DV, 1.3 mL of blood were expelled into an uncapped micro ethylenediaminetetraacetic acid tube (Sarstedt Inc, Newton, North Carolina)^14^ and the remaining blood was placed into an uncapped red top vacutainer tube containing clot activator (BD, Franklin Lakes, New Jersey).

For fPIVC samples, the area over a cephalic vein was clipped and sterilely prepared and a 20‐gauge, 1.25″ over‐the‐needle catheter (Terumo Medical Corporation, Somerset, New Jersey) was placed and secured with tape. Before flushing with saline, a 6‐mL syringe was attached to the catheter hub and 4 to 6 mL of blood were withdrawn and placed into tubes as previously described. If blood flow was slow, 2 syringes were used to collect 3 mL at a time instead. To assist blood collection, the vein proximal to the fPIVC was occluded with pressure from a finger.

For hPIVC, patency was assessed with a saline flush 24 ± 4 hours from time of placement. Blood collection from hPIVC was similar to a previously described study technique.^15^ All IV fluids and medications administered via the hPIVC were discontinued for 5 minutes before sampling. The *t*‐set clamp was adjusted to be as close as possible to the *t*‐set hub. After a 1 mL waste sample of blood was removed and discarded, blood for SB and CBC was collected as described above for fPIVC except that a 20‐gauge needle was attached to the syringe(s) and inserted into the injection port. Waste sample volume was determined by using saline to measure dead space from a 20‐gauge, 1.25″ catheter and attached *t*‐set hub. This volume (0.23 mL) was multiplied by 300% and rounded up to obtain a waste volume of 1 mL.^15^

### 2.3 Laboratory analysis

Samples were submitted to the in‐hospital clinical pathology laboratory within 30 minutes after collection. Trained laboratory staff processed samples by routine methods for SB and CBC analysis. Samples from each pair were processed and analyzed together. Blood cell counts were assessed using an Advia 2120 Hematology Analyzer (Siemens Healthcare Diagnostics, Tarrytown, New York) and SB panels were performed using an AU480 biochemistry analyzer (AU480) (Beckman Coulter, Inc, Tokyo, Japan). Values below the linearity of the AU480 were excluded. Values above the linearity of the AU480 were reported after dilution. One author (S. S. K. Beatty, a clinical pathologist), masked to sample source, performed a manual blood film review of all hematology slides to confirm the leukocyte differential counts (DIFF) and describe cell morphology.

Analytes from SB included in statistical comparisons were: alkaline phosphatase (ALP), alanine aminotransferase (ALT), aspartate aminotransferase (AST), total bilirubin (TBIL), total protein (TP), albumin (ALB), total calcium (CA), phosphorus (PHOS), creatinine (CREA), blood urea nitrogen (BUN), GLU, cholesterol (CHOL), magnesium (MG), sodium (NA), K, chloride (CL), and HCO_3_.

Quantitative values, ranging from 0 to 6, for lipemia, icterus, and hemolysis (LIH) were provided as part of the AU480 output. These were converted to binary values of 0 (AU480 output of 0) or 1 (AU480 output of 1‐6) to indicate absence or presence, respectively, for statistical analysis. If quantitative values were not available, the lab technician's gross assessment (absence or presence of LIH) was used. Missing data points were excluded.

Evaluated CBC analytes included PCV, hematocrit (HCT), hemoglobin (HB), red blood cell count (RBC), mean corpuscular volume (MCV), mean corpuscular hemoglobin concentration (MCHC), hemoglobin distribution width (HDW), red blood cell distribution width (RDW), total platelet count (PLT), mean platelet volume (MPV), leukocyte count (WBC), plasma heat precipitated proteins (HPP), nucleated red blood cells (nRBC), red blood cell morphology, platelet morphology, leukocyte morphology, and leukocyte differential counts (DIFF) including neutrophil count (NEUT), band neutrophil count (BAND), lymphocyte count (LYMPH), monocyte count (MONO), eosinophil count (EOS), and basophil count (BASO). For platelets, automated PLT was reported unless thrombocytopenia prompted a manual estimate. In those cases, the mid‐point of the estimated range was reported. If there was clumping noted with a normal or increased automated PLT, the automated PLT was still reported as a minimum value for PLT. On blood smear review, platelets were characterized as “normal/adequate,” “decreased,” or “increased,” as well as “clumped” or “not clumped.” For statistical analysis, red blood cell morphology changes, leukocyte toxicity, and large or clumped platelets were converted to binary values (0 for absence or 1 for presence of the morphologic change). To be considered “present,” the abnormality had to have been noted at least once per every other 100× microscopic field. Acanthocytes, echinocytes, and keratocytes were also reported on a conventional 1+ to 4+ scale for statistical analysis.

Quality control assessment of analyzer variability and ongoing performance were assessed as described in Data S1, Supporting Information.

### 2.4 Statistical analysis

Descriptive statistics were reported for each analyte, demographic data, and number of hours between hPIVC placement and sampling. Shapiro‐Wilk test was used to assess whether measurements of each analyte (expected non‐Gaussian) and the differences between paired samples (expected Gaussian) were normally distributed. Normality was rejected for *P* ≤ .05 for Shapiro‐Wilk or if coefficient of skewness or coefficient of kurtosis had *P* ≤ .05. Commercially available software was used for analysis (MedCalc, MedCalc Software Ltd, Ostend, Belgium).

Percentage difference plots, inspired by Bland‐Altman plots, were used to assess for significant differences between sampling methods. The difference between sample pair values divided by the mean of the values was calculated for each sample pair. Differences between sample pair values were classified as significant if greater than previously published cTEa values applied as the bounds of acceptable agreement.^16^, ^17^

Bland‐Altman analysis was also used to assess bias for measured analytes. For analytes with normally distributed differences, bias was estimated from the mean of the differences using a paired samples *t* test. For analytes with non‐normally distributed differences, bias was estimated from the median of the differences using a Wilcoxon signed rank sum test. A *P*‐value ≤.05 was considered significant. Additionally, constant and proportional bias were estimated using Passing‐Bablok regression analysis. Constant bias was considered statistically significant if the 95% confidence intervals for the intercept did not include 0. Proportional bias was considered statistically significant if the 95% confidence intervals for the slope did not include 1. Statistically significant biases were reviewed subjectively to determine clinical significance.

McNemar's test was utilized to assess for differences in abnormalities reported as being present or absent. Wilcoxon signed rank test was utilized to evaluate for differences in the degree of reported acanthocytes, echinocytes, and keratocytes. A *P*‐value ≤.05 was considered significant.

Sample size was determined by previously described recommendations for adequate power for Bland‐Altman analysis (n ≥ 59 to allow for 1 disagreeing pair when *α* = .05 and *β* = 80%) and Passing‐Bablok regression (n ≥ 50).^18^, ^19^

## 3 RESULTS

### 3.1 Arm 1 (fPIVC sampling)

#### 3.1.1 Study group

A total of 72 dogs were enrolled. Eleven were excluded because of inability to place PIVC (n = 4), placement of PIVC without an author present (n = 3), inability to draw adequate fPIVC samples (n = 2), inappropriate sample handling (n = 1), and inability to perform DV (n = 1). For 1 dog, the CBC samples clotted after collection and were excluded. This left 61 paired SBs and 60 paired CBCs available for analysis. Saved slides from the CBC were missing in 7 cases, and because these could therefore not be reviewed by the study author, they were excluded from analysis of manually confirmed results such as DIFF and morphology (leaving 53 paired samples for those). Two paired ALB values and 1 paired TP value were excluded as they were lower than analyzer linearity (leaving 59 and 60 sample pairs, respectively).

The final 61‐dog study group included 27 spayed females, 7 intact females, 20 neutered males, and 7 intact males. Body weights ranged from 8.3 to 68.0 kg (median 26.0 kg) and age ranged from 0.3 to 14.8 years (median 8.8 years). A variety of breeds were represented. Forty‐six dogs were clinically ill, 7 had nonurgent conditions necessitating elective procedures (such as surgical correction of an angular limb deformity), and 8 were healthy and undergoing routine procedures such as ovariohysterectomy.

#### 3.1.2 Biochemistry

**FIGURE 1:**
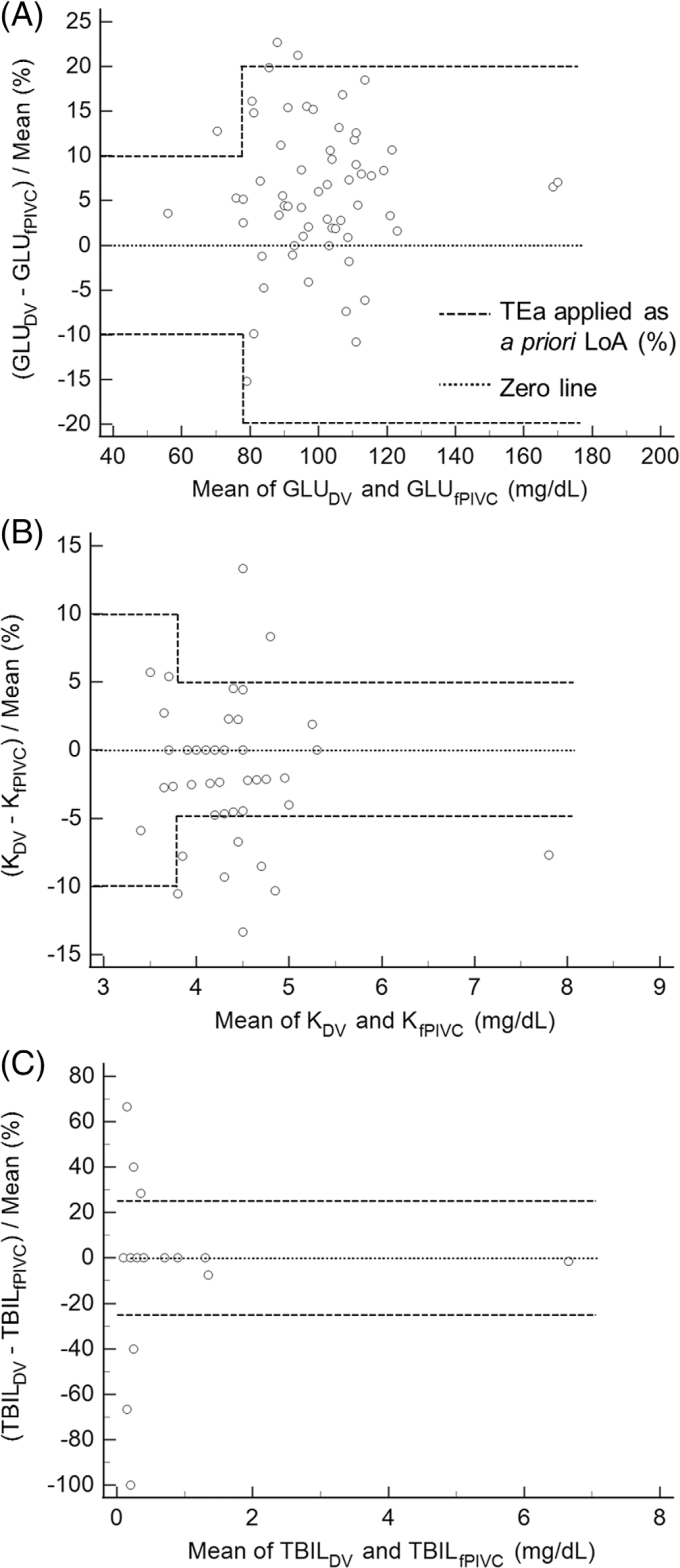
Selected percentage plots, inspired from Bland‐Altman plots, of serum biochemistry data with a priori bounds of acceptable agreement based on cTEa listed in Table 1 applied.^16^ The zero line indicates 0% difference between the paired samples. These selected examples include analytes for which there were multiple paired sample pairs with disagreement including (A) glucose (GLU_fPIVC_), (B) potassium (K_fPIVC_), and (C) total bilirubin (TBIL_fPIVC_). DV, direct venipuncture; fPIVC, freshly placed peripheral intravenous catheter

The percentage of sample pairs within cTEa bounds for each analyte is reported in Table 1. Analytes with at least 1 disagreeing sample pair included ALP, AST, PHOS, BUN, GLU (Figure 1A), K (Figure 1B), TBIL (Figure 1C), and TCO2. Each disagreeing sample pair was subjectively assessed individually to determine the effect on clinical decision‐making (Table S3). There were no decision‐altering pairs for ALP or TBIL. Of disagreeing sample pairs, results for 1 BUN pair (184 vs. 144 mg/dL, 24%) and 1 PHOS pair (65.1 vs. 54.1 mg/dL, 18%) were reported after dilution. Descriptive statistics for biochemistry data and linearity of the AU480 are reported in Table S4. Normality assessments for biochemistry data are reported in Table S5.

**TABLE 1:** Percentage of sample pairs within consensus total allowable error for serum biochemistry analytes^16^

Analyte	cTEa (%)^16^	RI	Units	Paired samples within cTEa	
				Arm 1 (fPIVC)	Arm 2 (hPIVC)
ALP	20	7‐116	IU/L	60/61 (98%)	60/61 (98%)
ALT	25	23‐93	IU/L	61/61 (100%)	61/61 (100%)
AST	30	23‐53	IU/L	55/61 (90%)	58/61 (95%)
TBIL	25	0.1‐0.4	mg/dL	50/61 (82%)	44/61 (72%)
TP	10	5.0‐7.4	g/dL	60/60 (100%)	61/61 (100%)
ALB	15	2.6‐3.9	g/dL	59/59 (100%)	61/61 (100%)
CA	10	8.7‐10.4	mg/dL	61/61 (100%)	60/61 (98%)
PHOS	20 (below RI); 15 (within or above RI)	2.2‐4.8	mg/dL	58/61 (95%)	61/61 (100%)
CREA	20	0.6‐1.5	mg/dL	61/61 (100%)	60/61 (98%)
BUN	15 (below RI); 12 (within or above RI)	7‐27	mg/dL	58/61 (95%)	57/61 (93%)
GLU	10 (below RI); 20 (within or above RI)	78‐124	mg/dL	58/61 (95%)	58/61 (95%)
CHOL	20	102‐340	mg/dL	61/61 (100%)	61/61 (100%)
MG	15	1.7‐2.4	mg/dL	61/61 (100%)	59/61 (97%)
NA	5	142‐151	mEq/L	61/61 (100%)	61/61 (100%)
K	10 (below RI); 5 (within or above RI)	3.8‐5.0	mEq/L	50/61 (82%)	54/61 (89%)
CL	5	108‐117	mEq/L	61/61 (100%)	61/61 (100%)
HCO_3_	15	16‐24	mEq/L	58/61 (95%)	55/61 (90%)

Abbreviations: ALB, albumin; ALP, alkaline phosphatase; ALT, alanine aminotransferase; AST, aspartate aminotransferase; BUN, blood urea nitrogen; CA, total calcium; CHOL, cholesterol; CL, chloride; CREA, creatinine; cTEa, consensus total allowable error; fPIVC, freshly placed peripheral intravenous catheter; GLU, glucose; HCO_3_, bicarbonate; hPIVC, peripheral intravenous catheter in a hospitalized patient; K, potassium; MG, total magnesium; NA, sodium; PHOS, phosphorus; RI, reference interval; TBIL, total bilirubin; TP, total protein.

Bias between sampling methods is reported in Table 2. There was statistically significant bias for several analytes, but only the bias for glucose was potentially clinically important.

**TABLE 2:** Bias between sampling methods for serum biochemistry analytes

Analyte	Units	Arm 1 (fPIVC)				Arm 2 (hPIVC)			
		Bland‐Altman		Passing‐Bablok		Bland‐Altman		Passing‐Bablok	
		Bias (CI)	*P*‐value	Constant bias (CI)	Proportional bias (CI)	Bias (CI)	*P*‐value	Constant bias (CI)	Proportional bias (CI)
ALP	IU/L	**−2.00 (−3.62, −1.00)** ^a^	<0.01	**−1.04 (−1.88, −0.02)** ^b^	**1.04 (1.03, 1.05)** ^b^	**1.00 (0.00, 2.00)** ^a^	<0.01	−0.58 (−1.22, 0.45)	0.99 (0.98, 1.00)
ALT	IU/L	**−1.00 (−1.00, −1.00)** ^a^	<0.01	**1.00 (0.17, 1.00)** ^b^	1.00 (0.17, 1.00)	**1.00 (0.00, 1.00)** ^a^	<0.01	−0.58 (−1.00, 0.33)	0.99 (0.97, 1.00)
AST	IU/L	−1.00 (−2.62, 0.62)	0.09	−0.30 (−2.73, 1.00)	1.04 (1.00, 1.09)	**2.00 (1.00, 4.00)** ^a^	<0.01	−1.00 (−2.00, 0.48)	0.98 (0.95, 1.00)
TBIL	mg/dL	0.00 (0.00, 0.00)	0.86	0.00 (0.00, 0.00)	1.00 (1.00, 1.00)	0.00 (0.00, 0.00)	0.50	0.00 (0.00, 0.00)	1.00 (1.00, 1.00)
TP	g/dL	**−0.20 (−0.20, −0.10)** ^a^	<0.01	0.20 (−0.29, 0.20)	1.00 (1.00, 1.08)	**0.10 (0.10, 0.20)** ^a^	<0.01	−0.10 (−0.56, 0.22)	1.00 (0.94, 1.08)
ALB	g/dL	**−0.06 (−0.08, −0.04)** ^c^	<0.01	0.02 (−0.13, 0.14)	1.02 (0.98, 1.07)	**0.05 (0.02, 0.07)** ^a^	<0.01	−0.09 (−0.22, 0.02)	1.02 (0.98, 1.06)
CA	mg/dL	**−0.10 (−0.16, −0.10)** ^a^	<0.01	0.10 (−0.74, 0.10)	1.00 (1.00, 1.09)	**0.10 (0.10, 0.20)** ^a^	<0.01	**−0.10 (−0.97, −0.10)** ^b^	1.00 (1.00, 1.09)
PHOS	mg/dL	**−0.10 (−0.16, −0.04)** ^a^	<0.01	**0.10 (0.10, 0.38)** ^b^	1.00 (0.92, 1.00)	**0.05 (0.01, 0.10)** ^c^	0.03	0.01 (−0.10, 0.27)	0.98 (0.92, 1.00)
CREA	mg/dL	**0.02 (0.00, 0.03)** ^a^	<0.01	−0.02 (−0.02, 0.01)	1.00 (0.97, 1.00)	**0.02 (0.01, 0.03)** ^a^	<0.01	0.01 (−0.02, 0.03)	**0.96 (0.93, 0.99)** ^b^
BUN	mg/dL	0.00 (0.00, 0.00)	0.59	0.00 (0.00, 0.00)	1.00 (1.00, 1.00)	0.00 (0.00, 0.00)	0.06	0.00 (0.00, 0.00)	1.00 (1.00, 1.00)
GLU	mg/dL	**5.89 (3.94, 7.83)** ^c^	<0.01	0.94 (−10.65, 11.17)	0.94 (0.83, 1.05)	**4.00 (3.00, 5.00)** ^a^	<0.01	4.56 (−4.00, 13.5)	0.92 (0.83, 1.00)
CHOL	mg/dL	**−8.00 (−9.00, −5.38)** ^a^	<0.01	−1.02 (−6.67, 3.12)	**1.04 (1.01, 1.06)** ^b^	**4.00 (2.38, 5.00)** ^a^	<0.01	−0.83 (−6.73, 5.46)	0.99 (0.96, 1.01)
MG	mg/dL	**0.00 (−0.10, 0.00)** ^a^	<0.01	0.00 (0.00, 0.00)	1.00 (1.00, 1.00)	**0.00 (0.00, 0.06)** ^a^	0.02	0.00 (0.00, 0.00)	1.00 (1.00, 1.00)
NA	mEq/L	**0.28 (0.01, 0.58)** ^c^	0.04	0.30 (−15.24, 13.21)	1.00 (0.91, 1.10)	0.00 (−0.22, 0.21)	0.98	0.10 (−11.04, 11.04)	1.00 (0.93, 1.08)
K	mEq/L	**−0.10 (−0.10. 0.00)** ^a^	0.04	0.10 (−0.49, 0.40)	1.00 (0.92, 1.13)	0.10 (0.00, 0.10)	0.19	−0.10 (−0.10, 0.48)	1.00 (0.87, 1.00)
CL	mEq/L	**0.61 (0.35, 0.87)** ^c^	<0.01	2.14 (−3.87, 8.89)	0.98 (0.91, 1.03)	**−0.30 (−0.76, −0.20)** ^a^	<0.01	−1.67 (−9.41, 5.00)	1.02 (0.96, 1.09)
HCO_3_	mEq/L	0.00 (−0.62, 1.00)	0.06	0.00 (−2.92, 0.00)	1.00 (1.00, 1.15)	**1.00 (0.38, 1.00)** ^a^	<0.01	**−1.00 (−4.17, −1.00)** ^b^	1.00 (1.00, 1.17)

Abbreviations: ALB, albumin; ALP, alkaline phosphatase; ALT, alanine aminotransferase; AST, aspartate aminotransferase; BUN, blood urea nitrogen; CA, total calcium; CHOL, cholesterol; CI, confidence interval 95%; CL, chloride; CREA, creatinine; fPIVC, freshly placed peripheral intravenous catheter; GLU, glucose; HCO3, bicarbonate; hPIVC, peripheral intravenous catheter in a hospitalized patient; K, potassium; MG, total magnesium; NA, sodium; PHOS, phosphorus; TBIL, total bilirubin; TP, total protein.

^a^ Indicates that bias (median of the differences for non‐normally distributed differences) determined by Bland‐Altman analysis was statistically significant (*P* ≤ .05) by a Wilcoxon signed rank sum test. Bias is reported with 95% confidence intervals from the median. Significant values are bolded.

^b^ Indicates that bias determined by Passing‐Bablok regression was considered statistically significant because the 95% confidence intervals did not include 0 (for constant bias) or 1 (for proportional bias). Significant values are bolded.

^c^ Indicates that bias (mean of the differences for normally distributed differences) determined by Bland‐Altman analysis was statistically significant (*P* ≤ .05) by a paired samples *t* test. Bias is reported with 95% confidence intervals from the mean. Significant values are bolded.

#### 3.1.3 Hematology

The percentage of sample pairs within cTEa bounds for each analyte is reported in Table 3. There was at least 1 disagreeing sample pair for almost all CBC parameters, but only the DIFF had less than 95% samples within cTEa (Table S6). Subjectively, very few differences would have affected clinical decision‐making. Descriptive statistics for CBC data are reported in Table S7. Normality assessments for CBC data are reported in Table S8.

**TABLE 3:** Percentage of sample pairs within consensus total allowable error for CBC analytes^17^

Analyte	cTEa (%)^17^	RI	Units	Paired Samples Within cTEa	
				Arm 1 (fPIVC)	Arm 2 (hPIVC)
WBC	15	5–13	K/μL	51/53 (96%)	50/52 (96%)
RBC	10	5.7‐8.3	M/μL	60/60 (100%)	47/60 (78%)
HB	10	14‐20	g/dL	59/60 (98%)	47/60 (78%)
HCT	10	40‐56	%	59/60 (98%)	48/60 (80%)
PCV	10	40–56	%	58/60 (97%)	50/59 (85%)
MCV	7	64‐74	fL	60/60 (100%)	60/60 (100%)
MCHC	10	33‐38	g/dL	60/60 (100%)	59/60 (98%)
PLT	20	134‐396	K/μL	52/53 (98%)	49/52 (94%)
NEUT	15	2.7‐8.9	K/μL	47/53 (89%)	25/52 (48%)
LYMPH	15	0.9‐3.4	K/μL	9/53 (17%)	11/52 (21%)
MONO	NCR (below RI); 60 (within RI); 50 (above RI)	0.1‐0.8	K/μL	35/53 (66%)	33/52 (63%)
EOS	90 (below RI); 50 (within and above RI)	0.1‐1.3	K/μL	24/53 (45%)	19/52 (37%)

Abbreviations: cTEa, consensus total allowable error; EOS, eosinophil count; fPIVC, freshly placed peripheral intravenous catheter; HB, hemoglobin; HCT, hematocrit; hPIVC, peripheral intravenous catheter in a hospitalized patient; LoA, limits of agreement; LYMPH, lymphocyte count; MCHC, mean corpuscular hemoglobin concentration; MCV, mean corpuscular volume; MONO, monocyte count; NCR, not clinically relevant; NEUT, neutrophil count; PLT, total platelet count; RBC, red blood cell count; RI, reference interval; WBC, leukocyte count.

Bias between sampling methods is reported in Table 4. There was statistically significant bias for several analytes, none of which were considered clinically important.

**TABLE 4:** Bias between sampling methods for CBC analytes

Analyte	Units	Arm 1 (fPIVC)				Arm 2 (hPIVC)			
		Bland‐Altman		Passing‐Bablok		Bland‐Altman		Passing‐Bablok	
		Bias (CI)	*P*‐value	Constant bias (CI)	Proportional bias (CI)	Bias (CI)	*P*‐value	Constant bias (CI)	Proportional bias (CI)
WBC	K/μL	0.03	0.78	0.16 (−0.24, 0.46)	0.98 (0.95, 1.03)	0.08 (−0.17, 0.33)	0.39	0.10 (−0.40, 0.61)	0.98 (0.95, 1.03)
RBC	M/μL	**−0.16 (−0.22, −0.10)** ^a^	<0.01	0.08 (−0.40, 0.50)	1.01 (0.95, 1.09)	**0.16 (0.10, 0.25)** ^b^	<0.01	−0.16 (−0.56, 0.30)	1.00 (0.92, 1.07)
HB	g/dL	**−0.39 (−0.51, −0.26)** ^a^	<0.01	0.18 (−0.87, 0.81)	1.01 (0.97, 1.08)	**0.45 (0.20, 0.61)** ^b^	<0.01	−0.45 (−1.26, 1.00)	1.00 (0.89, 1.06)
HCT	%	**−1.40 (−1.92, −0.69)** ^b^	<0.01	−0.04 (−3.12, 2.64)	1.03 (0.97, 1.11)	**0.95 (0.40, 1.62)** ^b^	<0.01	−0.62 (−3.64, 2.44)	0.99 (0.91, 1.07)
PCV	%	**−1.00 (−1.06, 0.00)** ^b^	<0.01	1.00 (−2.35, 1.00)	1.00 (1.00, 1.08)	**1.00 (0.00, 2.00)** ^b^	<0.01	−1.00 (−1.00, 3.44)	1.00 (0.89, 1.00)
MCV	fL	**−0.27 (−0.39, −0.18)** ^a^	<0.01	0.20 (−2.15, 2.15)	1.00 (0.97, 1.04)	**−0.15 (−0.21, 0.00)** ^b^	<0.01	0.15 (−1.43, 2.45)	1.00 (0.97, 1.02)
MCHC	g/dL	**0.16 (0.02, 0.30)** ^a^	0.02	1.72 (−3.10, 6.89)	0.95 (0.80, 1.08)	−0.10 (−0.30, 0.11)	0.23	−2.83 (−9.96, 2.80)	1.08 (0.92, 1.29)
RDW	%	0.00 (0.00, 0.00)	0.82	0.00 (0.00. 0.38)	1.00 (0.97, 1.00)	0.00 (0.00, 0.01)	0.16	0.00 (0.00, 0.00)	1.00 (1.00, 1.00)
MPV	fL	−0.15 (−0.50, 0.00)	0.13	0.79 (−0.41, 2.03)	0.95 (0.84, 1.06)	0.00 (−0.20, 0.10)	0.09	−0.20 (−1.02, 0.37)	1.02 (0.97, 1.10)
PLT	K/μL	−2.00 (−8.00, 4.00)	0.79	−3.75 (−14.08, 8.12)	1.02 (0.98, 1.06)	**9.00 (4.47, 16.14)** ^b^	<0.01	−3.07 (−12.16, 6.72)	0.97 (0.92, 1.02)
TS	g/dL	**−0.14 (−0.20, −0.08)** ^a^	<0.01	0.10 (−0.26, 0.56)	1.00 (0.94, 1.05)	**0.10 (0.05, 0.30)** ^b^	<0.01	−0.1 (−0.1, 0.56)	1.00 (0.89, 1.00)
NEUT	K/μL	0.08 (−0.16, 0.47)	0.29	0.14 (−0.20, 0.43)	0.97 (0.92, 1.02)	0.07 (−0.26, 0.41)	0.66	−0.45 (−1.13, 0.02)	1.04 (0.99, 1.10)
BAND	K/μL	0.00 (0.00, 0.00)	0.61	0.00 (0.00, 0.00)	0.78 (0.45, 1.57)	0.00 (0.00, 0.00)	0.09	0.00 (0.00, 0.00)	**0.43 (0.14, 0.80)** ^c^
LYMPH	K/μL	−0.10 (−0.22, 0.13)	0.61	−0.10 (−0.43, 0.15)	1.20 (0.93, 1.43)	0.01 (−0.22, 0.24)	0.93	−0.10 (−0.52, 0.19)	1.10 (0.89, 1.40)
MONO	K/μL	**−0.09 (−0.20, 0.02)** ^b^	0.03	0.02 (−0.18, 0.10)	1.25 (0.88, 1.59)	−0.01 (−0.22, 0.07)	0.36	−0.07 (−0.41, 0.09)	1.13 (0.90, 1.63)
EOS	K/μL	0.00 (0.07, 0.00)	0.37	**0.06 (0.01, 0.13)** ^c^	0.89 (0.61, 1.46)	0.00 (−0.11, 0.04)	0.66	−0.04 (0.22, 0.05)	1.19 (0.73, 2.07)
HPP	g/dL	0.00 (0.00, 0.00)	0.78	0.00 (−0.08, 0.00)	1.00 (1.00, 1.33)	0.00 (0.00, 0.00)	0.45	0.00 (0.00, 0.00)	1.00 (1.00, 1.00)

Abbreviations: BAND, band neutrophil count; CI, confidence interval 95%; EOS, eosinophil count; fPIVC, freshly placed peripheral intravenous catheter; HB, hemoglobin; HCT, hematocrit; hPIVC, peripheral intravenous catheter in a hospitalized patient; HPP, heat precipitated proteins; LYMPH, lymphocyte count; MCHC, mean corpuscular hemoglobin concentration; MCV, mean corpuscular volume; MONO, monocyte count; MPV, mean platelet volume; NEUT, neutrophil count; PLT, total platelet count; RBC, red blood cell count; RDW, red cell distribution width; TS, total solids; WBC, leukocyte count.

^a^ Indicates that bias (mean of the differences for normally distributed differences) determined by Bland‐Altman analysis was statistically significant (*P* ≤ .05) by a paired samples *t* test. Bias is reported with 95% confidence intervals from the mean. Significant values are bolded.

^b^ Indicates that bias (median of the differences for non‐normally distributed differences) determined by Bland‐Altman analysis was statistically significant (*P* ≤ .05) by a Wilcoxon signed rank sum test. Bias is reported with 95% confidence intervals from the median. Significant values are bolded.

^c^ Indicates that bias determined by Passing‐Bablok regression was considered statistically significant because the 95% confidence intervals did not include 0 (for constant bias) or 1 (for proportional bias). Significant values are bolded.

A summary of blood cell morphologic characterization is reported in Table 5. There was a significant difference (*P* = .05) in the presence of acanthocytes between DV and fPIVC samples (more frequent acanthocytes in the fPIVC samples) when the data was reported on a 0 to 4+ scale, but this statistical significance did not persist when the data was converted to binary terms (presence or absence) (*P* = .06). There were no other statistically significant differences in red blood cell morphology. One sample pair had “normal” platelets in the DV sample and “increased” platelets in the fPIVC sample. The automated PLT in this dog was above the reference interval (RI) and had excellent agreement between DV and fPIVC samples (442 vs. 452 K/μL, respectively, error −2%). There were 7 paired samples with disagreeing leukocyte morphology changes not listed in Table 5. These differences included identification of reactive and intermediate‐sized lymphocytes (1 DV sample, 2 fPIVC samples), identification of vacuolated macrophages (3 DV samples), and identification of HB crystals and granular intermediate lymphocytes (1 fPIVC sample). Statistical analysis could not be performed for hypochromasia, smudge cells, or stomatocytes because there were no disagreeing pairs (Table 5), or for BASO or nRBC because of low frequencies of these cell types. There was 1 disagreeing pair for BASO (0 vs. 0.07 K/μL, error −200%), and there were 8 disagreeing pairs for nRBC with error ranging from −200% to 200%.

**TABLE 5:** Summary of red blood cell, white blood cell, and platelet morphologic characterization

Morphologic change	Arm 1 (fPIVC)			Arm 2 (hPIVC)		
	# of disagreeing pairs (out of 53)	Wilcoxon *P*‐value	McNemar *P*‐value	# of disagreeing pairs (out of 52)	Wilcoxon *P*‐value	McNemar *P*‐value
Acanthocytes	8	0.05^a^	0.06	8	0.16	0.29
Anisocytosis	6		0.69	1		1.00
Codocytes	2		0.50	0		
Echinocytes	13	0.64	1.00	12	1.00	0.69
Eccentrocytes	2		1.00	2		0.50
Howell‐Jolly Bodies	1		1.00	0		
Hypochromia	0			1		1.00
Keratocytes	13	0.17	0.15	13	0.81	0.77
Polychromia	2		1.00	1		1.00
Rouleaux	6		1.00	7		1.00
Schistocytes	3		1.00	1		1.00
Smudge Cells	0			0		
Stomatocytes	0			0		
Leukocyte Toxicity	1		1.00	5		0.06
Hypersegmented NEUT	3		1.00	0		
Large PLT	5		0.38	3		0.25
Clumped PLT	11		1.00	6		1.00

Abbreviations: fPIVC, freshly placed peripheral intravenous catheter; hPIVC, peripheral intravenous catheter in a hospitalized patient; NEUT, neutrophil count; PLT, platelet.

^a^ *P*‐value ≤.05 is statistically significant.

#### 3.1.4 Lipemia, icterus, and hemolysis

Sixty‐one sample pairs were analyzed. There were no significant differences in the presence of hemolysis or lipemia between DV and fPIVC samples. Only 1 sample pair demonstrated icterus, so statistical comparison was not possible, but this dog had icterus in both DV and fPIVC samples.

### 3.2 Arm 2 (hPIVC sampling)

#### 3.2.1 Study group

A total of 76 dogs were enrolled. Of these, 15 dogs were excluded for: inability to draw an adequate hPIVC sample (n = 13), inappropriate sample handling (n = 1), and inability to perform DV (n = 1). For 1 dog, the CBC samples clotted after collection and were excluded. This left 61 paired SBs and 60 paired CBCs available for analysis. Slides were missing for 8 cases, leaving 52 paired samples for analysis of manually confirmed results. Manual PCV data was missing for 1 dog (leaving 59 paired samples).

The final 61‐dog study group included 26 spayed females, 7 intact females, 24 neutered males, and 4 intact males. A variety of breeds were represented. Body weights ranged from 8.1 to 55.7 kg (median 21.7 kg) and age ranged from 0.3 to 14.5 years (median 5.0 years). Forty‐one dogs were clinically ill, 13 had nonurgent conditions necessitating elective procedures, and 7 were healthy and undergoing routine procedures. Median time between hPIVC placement and sampling was 23 hours (range 20‐28 hours).

#### 3.2.2 Biochemistry

The percentage of sample pairs within cTEA bounds for each analyte is reported in Table 1. Analytes with at least 1 disagreeing sample pair included ALP, AST, TBIL, CA, CREA, BUN, GLU, MG, K, and HCO_3_ (Table S3). Each disagreeing sample pair was subjectively assessed individually to determine the effect on clinical decision‐making. There were no decision‐altering pairs for ALP, CA, CREA, BUN, MG, or K. Descriptive statistics for biochemistry data are reported in Table S4. Normality assessments for biochemistry data are reported in Table S5.

Bias between sampling methods is reported in Table 2. There was statistically significant bias for several analytes, but only bias for glucose was considered potentially clinically important.

#### 3.2.3 Hematology

The percentage of sample pairs within cTEA bounds for each analyte is reported in Table 3. There was at least 1 disagreeing sample pair for almost all CBC parameters (Table S6). The DIFF, RBC, HB, HCT, PCV, and PLT had less than 95% samples within cTEa. Subjectively, few differences would have affected clinical decision‐making. Descriptive statistics for CBC data are reported in Table S7. Normality assessments for CBC data are reported in Table S8.

**FIGURE 2:**
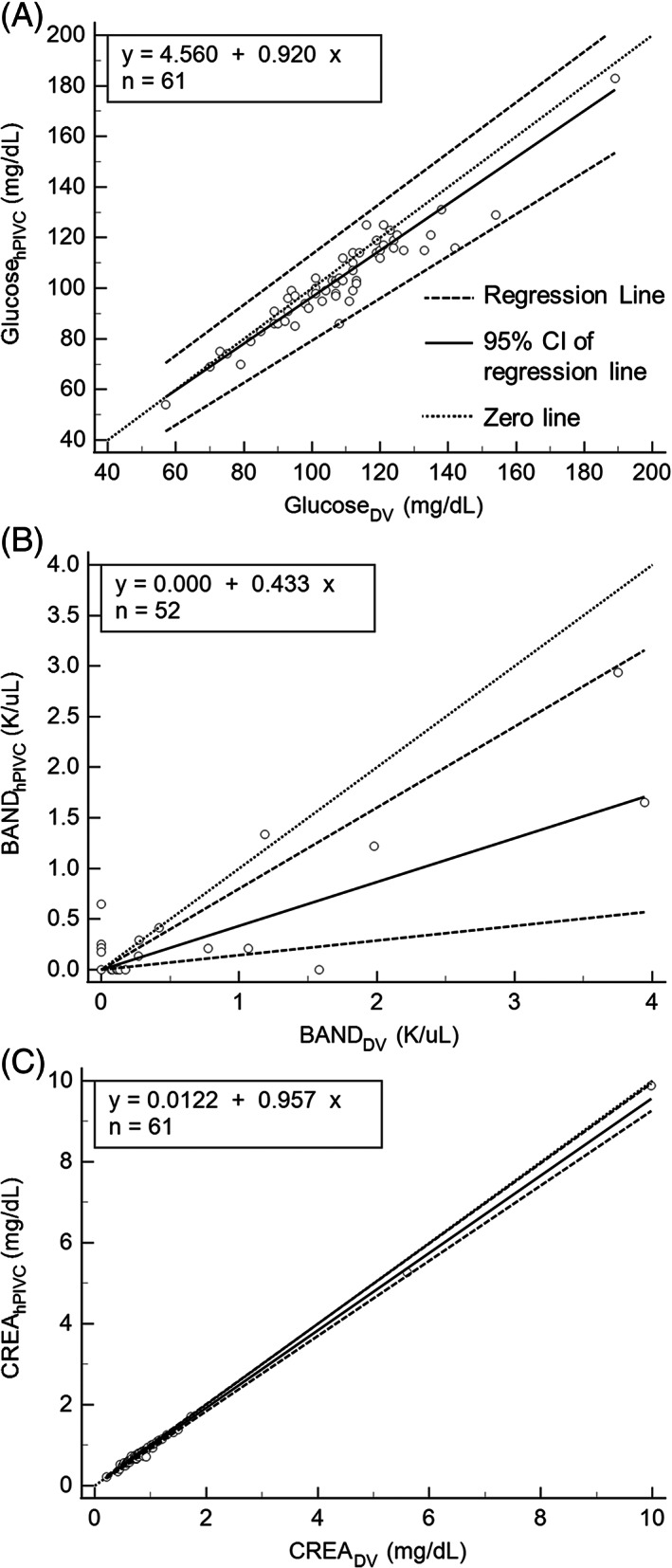
Selected Passing‐Bablok regression plots. The 0 line represents no difference between paired samples (y = x). A y‐intercept significantly different from 0 represents constant bias and a slope significantly different from 1 represents proportional bias. These selected examples include 1 analyte for which there was no significant bias (A) glucose (GLU_hPIVC_), and 2 for which there is statistically significant proportional bias including (B) band neutrophil count (BAND_hPIVC_), and (C) creatinine (CREA_hPIVC_). CI, confidence interval 95%; DV, direct venipuncture; hPIVC, peripheral intravenous catheter in a hospitalized dog

Bias between sampling methods is reported in Table 4. There was statistically, and possibly clinically, significant proportional bias (Passing‐Bablok regression) for BAND (Figure 2B). The remainder of the statistically significant biases were not deemed to be clinically important. There were no other statistically significant differences in red blood cell, leukocyte, or platelet morphology (Table 5). Statistical analysis could not be performed for codocytes, Howell‐Jolly bodies, smudge cells, stomatocytes because there were no disagreeing pairs (Table 5), or for BASO or nRBC because of low frequencies of these cell types. There were 6 disagreeing pairs for BASO with error ranging from −200% to 200%, and there were 8 disagreeing pairs for nRBC with error ranging from −200% to 200%.

#### 3.2.4 Lipemia, icterus, and hemolysis

Four data pairs were excluded because of missing data. There were no significant differences in presence of hemolysis, icterus, or lipemia between 57 DV and hPIVC sample pairs.

## 4 DISCUSSION

This study demonstrated few clinically important differences for most machine‐measured values for SB and CBC analytes between samples collected from dogs by DV and from PIVC at the time of placement or in place for approximately 1 day. However, there are occasional disagreements for certain analytes, and disagreement between samples was common for leukocyte differentials. Most observed disagreements were subjectively considered unlikely to lead to different clinical decisions. Bias related to sampling technique was uncommon, but when present, rarely caused disagreement between sample pairs and was subjectively considered clinically unimportant for most analytes. Evaluation of overall analytic performance excluded analyzer variability as a major source of variation between samples. However, we cannot rule out an effect related to sampling location (jugular versus cephalic). A previous study comparing some SB and CBC parameters between cephalic and jugular veins in dogs found small but statistically significant bias for higher CREA (0.03 mg/dL) and K (0.15 mEq/L) concentrations in jugular samples.^20^ In our study, there was statistically significant bias for CREA (fPIVC: 0.02; hPIVC: 0.02), which is consistent with this previous study. For K, there was statistically significant bias for fPIVC (−0.10) but not for hPIVC, which is not consistent with the previous study. Another study in dogs evaluating various CBC parameters found statistically significantly lower WBC (bias of −0.7 K/μL) and MONO (bias of −0.08 K/μL) in jugular vein samples compared to cephalic vein samples.^21^ In our study, there was no statistically significant bias for WBC. There was statistically significant bias for fPIVC MONO (−0.09) but not for hPIVC. To the authors' knowledge, comparison of cephalic and jugular values for other analytes in our study has not been performed.

Many disagreements between sample pairs were likely related to increments of measurement, particularly at the lower ends of measurement scales. For example, the SB analyte with the greatest proportion of sample pair differences outside cTEa was TBIL, including 18% and 28% for fPIVC (Figure 1C) and hPIVC groups, respectively. Because of the small magnitude of TBIL measurements in all of these sample pairs relative to the measured increments, differences are magnified when calculating error. A difference between TBIL measurement of 0.1 or 0.2 mg/dL is unlikely to change clinical decision‐making; however, the error for this sample pair is well outside cTEa of 25%.^16^ No sample pair difference for TBIL in this study would have changed clinical decision‐making. Thus, the authors consider DV and PIVC sampling methods as providing clinically equivalent TBIL results in this study group. The same conclusion is reached for BUN, another analyte in which differences between low magnitude numbers (ie, 7 and 8 mg/dL) are magnified when calculating error.

Values obtained via dilution are inherently more variable because of human and analyzer error. In this study, the majority of sample pairs that contained values obtained by dilution were within cTEa including (83% (10/12) for fPIVC and 100% (5/5) for hPIVC). The 2 disagreeing pairs included 1 fPIVC PHOS and 1 fPIVC BUN. If those sample pairs were removed from analysis, the percentage of sample pairs within the cTEA for PHOS and BUN for fPIVC both improved from 95% to 97%. Without a much larger number of paired samples with values in a range that would require dilution, it is difficult to determine if disagreements between samples are related to the sampling technique or error. Clinicians should always interpret specific number values obtained by dilution cautiously. However, these values are generally far enough outside the RI that initial clinical interpretation and decision making are unlikely to be different even if TEa is exceeded.

For SB, the only analytes with >5% of sample pair results outside cTEa that could not be explained by small increments of measurement or dilution were AST, K, and HCO_3_. There was no clinically significant constant or proportional bias for any of these, so it is difficult to develop theories to explain the disagreements. Of these, there were only 6 differences (2 for AST, 2 for K, and 3 for HCO_3_) out of 122 sample pairs for which, depending upon individual clinician interpretation, clinical decision‐making could have affected. The differences in AST resulted in 1 value being above the RI, which might have affected a decision about pursuing further investigation. The differences in potassium and HCO_3_ might have altered decisions about potassium supplementation or alkalinization treatments. Ultimately, PIVC sampling for these analytes is generally reliable, but confirming results for values that are close to either end of a RI or not expected to be abnormal should be considered.

For automated CBC analytes other than leukocyte differential, there were few sample pair differences outside cTEa. For the hPIVC group, >5% of sample pairs had a disagreement between RBC, HB, HCT, or PCV, and there was a slightly greater likelihood of sample pair disagreement for all CBC values except MCV when compared to fPIVC. An effect of prior fluid or medication administration through the PIVC cannot be entirely ruled out despite the 5‐minute period before sampling, but there was no bias consistent with dilution. Another explanation is that hPIVCs might have compromised lumens because of kinks or fibrin deposition, resulting in increased shear and fragmentation injury of RBCs. However, if this were the case, we would expect significant bias in certain values suggestive of hemolysis (increased HB, decreased RBC, decreased HCT, and decreased PCV) as well as increased frequency of hemolysis in hPIVC samples. Neither clinically important bias or differences in hemolysis were identified.

There were significant disagreements for DIFF between sampling methods for both fPIVC and hPIVC. If the poor agreement between DIFF was attributed solely to use of a PIVC, significant bias between sampling methods would be expected. It is more likely that the poor agreement is reflective of inadequate precision and high variability of the DIFF in general, as previously reported.^22^, ^23^, ^24^, ^25^ Protocols in this study (simultaneous preparation of blood smears from paired samples by the same technician and use of a single clinical pathologist to review all blood smears) were meant to reduce sources of variability, but do not eliminate intraobserver variability. In one study, the range for intraobserver coefficient of variation for BAND was 0% to 141%.^25^ Additionally, similar to some of the SB values, small differences between samples with small numbers of specific cell types can be magnified when calculating error.

There was statistically significant constant or proportional bias for multiple SB and CBC analytes in both fPIVC and hPIVC groups. However, apart from GLU and BAND, for values within or close to the RI, it appears that bias related to sampling technique was not a clinically important problem. However, the small number of samples with values that were well above the RI could have created relevant overestimation or underestimation of proportional bias.

**FIGURE 3:**
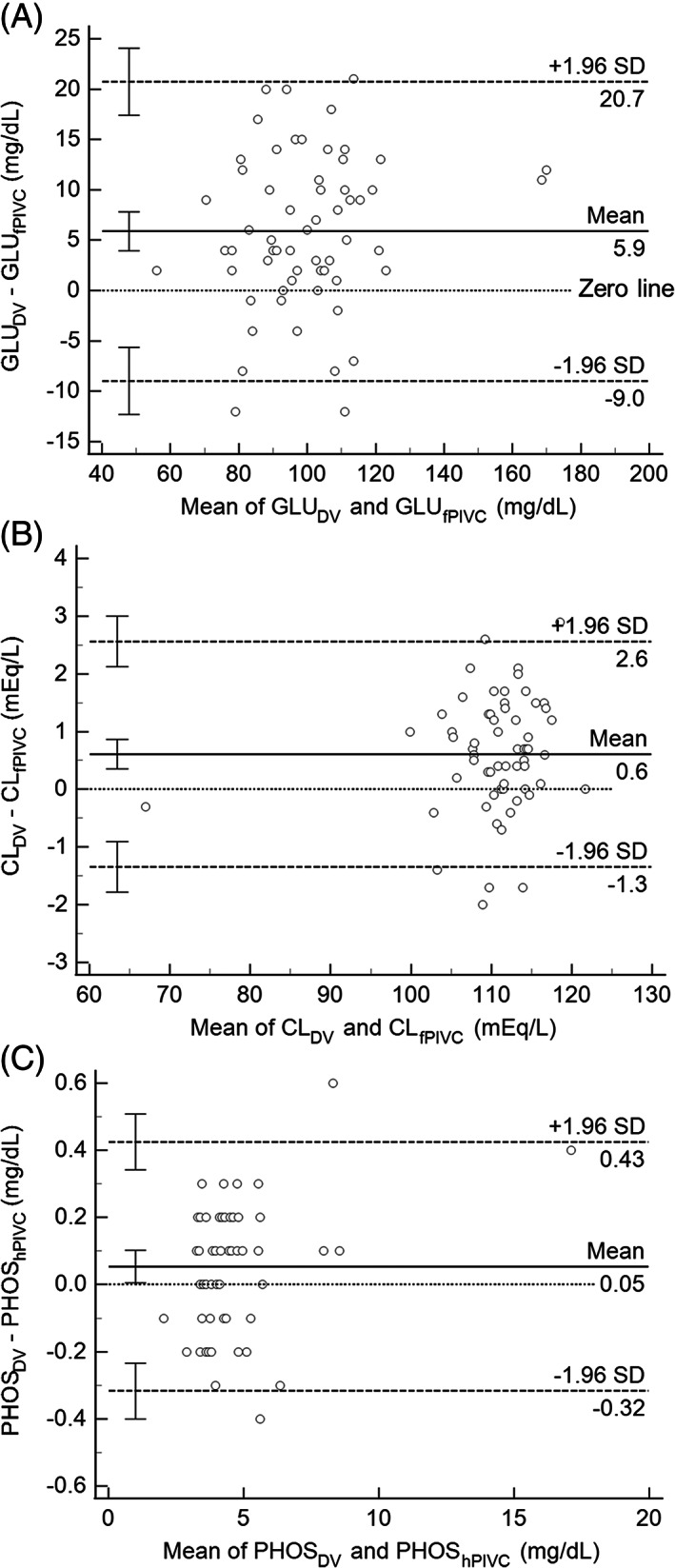
Selected Bland‐Altman plots. The limits of agreement (LoA) are calculated as 1.96 times the SD of the mean. The 0 line indicates 0 difference between the paired samples. The mean difference line is an estimate of bias between sampling methods. These selected examples include analytes for which there was statistically significant bias including (A) glucose (GLU_fPIVC_), (B) chloride (CL_fPIVC_), and (C) phosphorus (PHOS_hPIVC_). CI, confidence interval 95%; DV, direct venipuncture; fPIVC, freshly placed peripheral intravenous catheter; hPIVC, peripheral intravenous catheter in a hospitalized dog

There was statistically and potentially clinically significant bias for paired GLU measurements (Figures 2A and 3A). The DV sample was, on average, 4.0 mg/dL (hPIVC, Bland‐Altman) to 5.9 mg/dL (fPIVC, Bland‐Altman) higher than fPIVC and hPIVC samples. Whether this bias would be considered clinically important could be dependent upon the individual clinician or the dog's circumstances. In most cases, a difference of about 5 mg/dL would not change clinical decision‐making, especially if the glucose values are within or above the RI. However, this bias could alter clinical decision‐making for values at the lower end or below the RI, such as the 2 paired data points identified in the results section (fPIVC: 75 vs. 66 mg/dL, error 13%; hPIVC: 79 vs. 70 mg/dL, error 12%). It is unclear why GLU from the DV sample was consistently higher than fPIVC or hPIVC samples. In most previous human studies, there were no significant differences in GLU between sampling methods.^7^, ^8^, ^9^, ^10^ In a study comparing PIVC sampling to DV in children, however, PIVC samples were an average of 5 mg/dL higher in PIVC samples compared to DV samples and 22% of samples were outside acceptable limits for variation.^6^ In that study, fluids, including dextrose‐containing solutions, were only discontinued for 1 minute before sample collection. Another study of adult humans found GLU was 1.7 mg/dL higher on average in DV samples than PIVC samples 2 minutes after a saline bolus through the PIVC.^13^ The authors deemed glucose measurements between sampling methods were not equivalent. However, the reported laboratory error of 2.4 mg/dL was greater than the mean difference, making this data difficult to interpret.^13^

There was also statistically and possibly clinically significant proportional bias for BAND from hPIVC samples, with more BAND identified in DV than PIVC samples (Figure 2B). This bias was not found for fPIVC samples. The hPIVC BAND bias might be secondary to differences in the ability of these cells to pass through hPIVC because of kinks, fibrin deposition, or both. Differences in marginalization of BAND between jugular and cephalic veins are less likely because bias was not present for fPIVC, and differences in BAND concentrations between jugular and cephalic venous samples was not identified in a previous study of dogs.^21^

There were no significant differences in presence of lipemia or hemolysis between sampling methods. A limitation is that the AU480 is not validated to quantify the degree of lipemia, hemolysis, or icterus in canine samples. It is possible that a relationship between the degree of interfering substances, especially HB, could exist between sampling methods. However, this study was not designed to detect this difference. Another limitation was the inability to statistically analyze the effect of hemolysis on abnormal biochemistry values. This was because of the lack of validated quantitative LIH data and the low number of disagreeing pairs, which might have resulted in type II error.

Interestingly, the only significant difference in RBC morphology between DV and PIVC sampling methods was for acanthocytes from fPIVC samples. This statistically significant difference did not persist for hPIVC. It is possible that the fPIVC acanthocyte difference represents type I error, or the lack of acanthocyte difference for hPIVC represents type II error. While acanthocytes could form secondary to fragmentation injury as RBCs travel through the fPIVC, there were no other indications of increased shear forces.

It is difficult to determine an appropriate number of samples to adequately power a study utilizing Bland‐Altman analysis, but a higher number of paired samples provides more confidence for conclusions about whether there is clinically acceptable agreement.^18^ To maintain adequate power (when *α* = .05 and *β* = 80%) with 1 disagreeing sample pair, the recommended sample size is n ≥ 59.^18^ For the analytes with 2 to 3 pair sample disagreements in this study, a larger sample size might more cleanly determine whether we should conclude that the sampling methods are clinically equivalent or not.

There were several additional limitations to this study. There were a small number of values above or below the RI for each analyte, limiting the ability to extrapolate the reliability of PIVC sampling to markedly abnormal values. In particular, because of the study design, there were very few severely anemic or thrombocytopenic dogs. The findings of this study cannot be extrapolated to dogs with those conditions. Additionally, values outside of the linearity of the AU480 were excluded (if below) or diluted (if above). We attempted to address dilution concerns by evaluating every disagreeing serum biochemistry sample pair. Other limitations include the assessment of only 1 catheter size and 2 time points for PIVC sampling. It is possible that results could be affected by different catheter sizes, collection techniques or waste volumes, or longer time from placement. The missing CBC slides that resulted in exclusion of some blood cell morphology data was a further limitation.

In conclusion, the results of this study generally support the clinical utility of PIVC sampling for SB and CBC analyses under the conditions used in this study (fPIVC or hPIVC in place for approximately 24 hours). Uncommonly, results for a few analytes (GLU, AST, K, HCO_3_, RBC, HB, HCT, and PCV) might have differences between sampling methods that could be sufficient to alter clinical decision‐making, particularly for values fairly close to the low and high ends of the RI. There was poor agreement between sampling methods for leukocyte differential counts, but differences are unlikely to be solely, or even primarily, related to sampling method. Clinicians should consider verifying results with a DV sample if unexpected PIVC results might impact clinical decision‐making.

## CONFLICT OF INTEREST DECLARATION

Authors declare no conflict of interest.

## OFF‐LABEL ANTIMICROBIAL DECLARATION

Authors declare no off‐label use of antimicrobials.

## INSTITUTIONAL ANIMAL CARE AND USE COMMITTEE (IACUC) OR OTHER APPROVAL DECLARATION

Approved by the University of Florida IACUC, 201810519; and the University of Florida College of Veterinary Medicine Veterinary Hospital research review committee, 2019‐11.

## HUMAN ETHICS APPROVAL DECLARATION

Authors declare human ethics approval was not needed for this study.

## Supporting information


**Appendix S1** Supplementary tables.
