# Supplementary material for: Comparison of biochemical and hematologic values obtained via jugular venipuncture and peripheral intravenous catheters in dogs
Source: J Vet Intern Med. 2022 Aug 27;36(5):1628–40. doi: 10.1111/jvim.16518 (PMC9511059; doi:10.1111/jvim.16518)
Supplement: Supplementary file 1 — Appendix S1 Supplementary tables. [file JVIM-36-1628-s001.docx]

**Quality control assessment of analyzer variability and ongoing performance.**

**Methods:** Using the American Society of Veterinary Clinical Pathology guidelines, total error was determined for each analyte on both analyzers and verified as less than consensus values for total allowable error (cTEa).^16, 17^ Bias %, total observed error (TEobs), and Sigma values were then obtained and TEobs was compared to the cTEa. Ongoing performance was monitored every 24 hours on days patient samples were analyzed in the clinical pathology laboratory. Three levels of commercial quality control material (low, normal, and high) were used for hematology and two levels of quality control material (normal, high) were used for chemistry. Quality control material were used for calculation of a mean value, standard deviation, and coefficient of variation (CV). For this study, a 30-day period was utilized for evaluation. Additionally, a quality goal index (QGI) was calculated for Sigma values <6 to assist with interpretation.

**Results:** All observed total error values were within available cTEa for veterinary hematology and biochemistry (Supplementary Tables 1 and 2).^16, 17^ Sigma values for most analytes at most time points were >5-6, indicating excellent to world-class quality.^26^ Some analytes (CREA – level 3, GGT – level 2, WBC and RBC) had sigma values between 4-5 (indicating good quality) and some analytes (ALP, AST – level 2, BUN, TP, HCO3, NA, K, and CL) had sigma values between 3-4 (indicating marginal, yet acceptable quality).^26^ For analytes with sigma values between 3-4, QGIs were interpreted as follows: TP, HCO3 – level 2, NA, K, and CL were affected by imprecision. AST level 2, ALP, and HCO3 – level 3 were affected by inaccuracy. BUN was affected by both precision and inaccuracy.

**Supplementary Table 1.** Analyzer variability for biochemistry analytes.

| **Analyte** | **Level** | **CV (%)** | **Bias (%)** | **TE_obs_ (%)** | **cTEa (%)** | **Sigma** | **QGI** |
| --- | --- | --- | --- | --- | --- | --- | --- |
| ALP | 2-normal | 3.1 | 13.5 | 19.7 | 25 | 3.7 | 2.9 |
|  | 3-high | 2.9 | 14.2 | 20.0 | 25 | 3.7 | 3.2 |
| ALT | 2-normal | 2.2 | 12.7 | 17.1 | 25 | 5.5 | 3.8 |
|  | 3-high | 1.7 | 15.6 | 18.9 | 25 | 5.7 | 6.3 |
| AST | 2-normal | 2.8 | 21.6 | 27.2 | 30 | 3.0 | 5.2 |
|  | 3-high | 1.7 | 19.6 | 23.0 | 30 | 6.1 | - |
| TBIL | 2-normal | 1.0 | 2.7 | 4.7 | 25 | 21.8 | - |
|  | 3-high | 1.7 | 4.3 | 7.8 | 25 | 11.9 | - |
| TP | 2-normal | 2.4 | 1.4 | 6.3 | 10 | 3.5 | 0.4 |
|  | 3-high | 2.3 | 0.8 | 5.4 | 10 | 4.0 | 0.2 |
| ALB | 2-normal | 1.5 | 4.4 | 7.4 | 15 | 7.0 | - |
|  | 3-high | 1.4 | 3.2 | 6.0 | 15 | 8.5 | - |
| CA | 2-normal | 1.5 | 1.6 | 4.5 | 10 | 5.8 | 0.7 |
|  | 3-high | 1.4 | 1.6 | 4.5 | 10 | 5.8 | 0.8 |
| PHOS | 2-normal | 2.3 | 1.3 | 6.0 | 15 | 5.8 | 0.4 |
|  | 3-high | 2.6 | 0.5 | 5.6 | 15 | 5.7 | 0.1 |
| CREA | 2-normal | 3.1 | 2.7 | 9.0 | 20 | 5.5 | 0.6 |
|  | 3-high | 2.1 | 10.3 | 14.5 | 20 | 4.6 | 3.3 |
| BUN | 2-normal | 2.5 | 4.2 | 9.2 | 12 | 3.1 | 1.1 |
|  | 3-high | 2.1 | 3.3 | 7.4 | 12 | 4.2 | 1.1 |
| GLU | 2-normal | 1.3 | 0.3 | 2.9 | 20 | 14.7 | - |
|  | 3-high | 1.2 | 2.5 | 5.0 | 20 | 14.4 | - |
| CHOL | 2-normal | 1.4 | 4.6 | 7.4 | 20 | 11.2 | - |
|  | 3-high | 1.3 | 4.5 | 7.1 | 20 | 12.2 | - |
| MG | 2-normal | 2.1 | 4.8 | 8.9 | 20 | 7.4 | - |
|  | 3-high | 2.0 | 5.2 | 9.2 | 20 | 7.3 | - |
| NA | 2-normal | 1.1 | 1.3 | 3.5 | 5 | 3.3 | 0.8 |
|  | 3-high | 1.3 | 1.0 | 3.6 | 5 | 3.0 | 0.5 |
| K | 2-normal | 1.5 | 0.3 | 3.3 | 5 | 3.2 | 0.2 |
|  | 3-high | 1.4 | 0.5 | 3.4 | 5 | 3.2 | 0.3 |
| CL | 2-normal | 1.3 | 0.7 | 3.2 | 5 | 3.4 | <0 |
|  | 3-high | 1.4 | 0.5 | 3.1 | 5 | 3.4 | <0 |
| HCO_3_ | 2-normal | 5.3 | 1.0 | 11.7 | 20 | 3.5 | 0.1 |
|  | 3-high | 3.7 | 7.6 | 14.9 | 20 | 3.4 | 1.4 |

Abbreviations: ALB, albumin; ALP, alkaline phosphatase; ALT, alanine aminotransferase; AST, aspartate aminotransferase; BUN, blood urea nitrogen; CA, total calcium; CHOL, cholesterol; CL, chloride; CREA, creatinine; cTEa, consensus total allowable error; CV, coefficient of variation; GLU, glucose; HCO_3_, bicarbonate; K, potassium; MG, total magnesium; NA, sodium; PHOS, phosphorus; QGI, quality goal index; TBIL, total bilirubin; TE_obs_, observed total error; TP, total protein

**Supplementary Table 2.** Analyzer variability for CBC analytes.

| **Analyte** | **Level** | **CV (%)** | **Bias (%)** | **TE_obs_ (%)** | **cTEa (%)** | **Sigma** | **QGI** |
| --- | --- | --- | --- | --- | --- | --- | --- |
| WBC | 1-low | 4.7 | 0.7 | 10.1 | 20 | 4.1 | 0.2 |
|  | 2-normal | 5.1 | 1.4 | 11.5 | 20 | 3.7 | 0.3 |
|  | 3-high | 4.7 | 1.8 | 11.2 | 20 | 3.9 | 0.4 |
| RBC | 1-low | 1.6 | 1.6 | 4.8 | 10 | 4.1 | 1.2 |
|  | 2-normal | 1.5 | 1.9 | 5.0 | 10 | 4.0 | 1.0 |
|  | 3-high | 1.7 | 0.9 | 4.2 | 10 | 5.2 | 1.0 |
| HB | 1-low | 1.3 | 0.2 | 2.8 | 10 | 7.6 | - |
|  | 2-normal | 1.1 | 3.3 | 5.5 | 10 | 6.2 | - |
|  | 3-high | 0.9 | 2.7 | 4.5 | 10 | 8.1 | - |
| HCT | 1-low | 2.5 | 3.1 | 8.0 | 15 | 4.8 | 1.3 |
|  | 2-normal | 1.7 | 4.3 | 7.7 | 15 | 6.2 | - |
|  | 3-high | 1.7 | 3.5 | 6.9 | 15 | 6.9 | - |
| MCV | 1-low | 1.0 | 3.4 | 5.5 | 7 | 3.4 | 3.3 |
|  | 2-normal | 0.8 | 3.6 | 5.1 | 7 | 4.4 | 4.6 |
|  | 3-high | 0.7 | 3.9 | 5.2 | 7 | 4.7 | 5.7 |
| MCHC | 1-low | 1.5 | 5.6 | 8.6 | 10 | 2.9 | 3.6 |
|  | 2-normal | 2.3 | 2.6 | 7.2 | 10 | 3.2 | 1.1 |
|  | 3-high | 1.3 | 0.7 | 3.3 | 10 | 11.7 | - |
| PLT | 1-low | 4.1 | 7.4 | 15.5 | 20 | 3.1 | 1.8 |
|  | 2-normal | 2.4 | 9.0 | 13.8 | 20 | 4.6 | 3.8 |
|  | 3-high | 3.9 | 7.3 | 15.1 | 20 | 50.0 | - |

Abbreviations: cTEa, consensus total allowable error; CV, coefficient of variation; HB, hemoglobin; HCT, hematocrit; MCHC, mean corpuscular hemoglobin concentration; MCV, mean corpuscular volume; PLT, total platelet count; RBC, red blood cell count; QGI, quality goal index; TE_obs_, observed total error; WBC, leukocyte count

**Supplementary Table 3.** Serum biochemistry data pairs outside of consensus total allowable error.

| Analyte | Units | Arm 1 | | Arm 2 | |
| --- | --- | --- | --- | --- | --- |
|  |  | Sample pairs outside of cTEa | Specific Sample Pairs  (DV, fPIVC) | Samples pairs outside of cTEa | Specific Sample Pairs  (DV, hPIVC) |
| **ALP** | IU/L | 1 | (24, 17) | 1 | (48, 33) |
| **ALT** | IU/L | 0 | ^-^ | 0 | - |
| **AST** | IU/L | 6 | (28, 18); (26, 18); (26, 49); (22, 33); (28, 49); **(43, 62)**^* §^ | 3 | (28, 19); (33, 21); **(53, 85)**^§^ |
| **TBIL** | mg/dL | 11 | (0.3, 0.2); (0.1, 0.2); (0.2, 0.3); (0.3, 0.2); (0.4, 0.3); (0.4, 0.3); (0.3, 0.2); (0.1, 0.3); (0.2, 0.3); (0.2, 0.3); (0.2, 0.1) | 17 | (0.2, 0.1); (0.2, 0.3); (0.2, 0.1); (0.3, 0.2); (0.2, 0.3); (0.3, 0.4); (0.2, 0.1); (0.2, 0.3); (0.3, 0.2); (0.1, 0.2); (0.2, 0.3); (0.4, 0.2); (0.4, 0.3); (0.3, 0.2); (0.4, 0.2); (0.2, 0.4) |
| **TP** | g/dL | 0 | - | 0 | - |
| **ALB** | g/dL | 0 | - | 0 | - |
| **CA** | mg/dL | 0 | - | 1 | (9.6, 8.5) |
| **PHOS** | mg/dL | 3 | (2.3, 2.8); **(65.1, 54.1)**^§^;  **(1.6, 2.0)**^§^ | 0 | - |
| **CREA** | mg/dL | 0 | - | 1 | (0.92, 0.72) |
| **BUN** | mg/dL | 3 | (8, 7); (7, 8); **(184, 144)**^*§^ | 4 | (8, 7); (6, 5); (7, 6); (8, 7) |
| **GLU** | mg/dL | 3 | (104, 84); (98, 78); **(75, 66)**^§^ | 3 | (142, 116); (108, 86); **(79, 70)**^§^ |
| **CHOL** | mg/dL | 0 | - | 0 | - |
| **MG** | mg/dL | 0 | - | 2 | (1.8, 2.3); (2.6, 2.2) |
| **NA** | mEq/L | 0 | - | 0 | - |
| **K** | mEq/L | 11 | (4.3, 4.6); (5.0, 4.6); (4.2, 4.8); (7.5, 8.1); (4.1, 4.5); (5.0, 4.6); (4.8, 4.2); (4.6, 5.1); (4.5, 4.9) **(3.6, 4.0)**^§^; **(3.7, 4.0)**^§^ | 7 | (4.0, 3.8); (4.6, 4.3); (4.4, 4.7); (4.7, 4.3); (5.1, 4.6); (3.9, 4.2); (3.9, 4.2) |
| **CL** | mEq/L | 0 | - | 0 | - |
| **HCO_3_** | mEq/L | 3 | **(9, 6)**^§^; **(20, 15)**^§^; (20; 17) | 6 | (20, 17); (24, 20); (21, 18); **(17, 14)**^§^ |

 Abbreviations: ALB, albumin; ALP, alkaline phosphatase; ALT, alanine aminotransferase; AST, aspartate aminotransferase; BUN, blood urea nitrogen; CA, total calcium; CHOL, cholesterol; CL, chloride; CREA, creatinine; DV, direct venipuncture; fPIVC, freshly-placed peripheral

^*^Indicates that this sample pair required dilution because it was outside of the analyzer linearity.

^§^Indicates that the difference between the values in this sample pair was assessed to potentially affect clinical decision making. These sample pairs are in bold.

**Supplementary Table 4.** Descriptive statistics and analyzer linearity for serum biochemistry data.

| Analyte | Arm 1 | | Arm 2 | | RI |  | Units |
| --- | --- | --- | --- | --- | --- | --- | --- |
|  | DV  Median (range) | fPIVC  Median (range) | DV  Median (range) | hPIVC  Median (range) |  | AU480 Linearity |  |
| ALP | 81 (15-8948) | 80 (15-9661) | 93 (21-6733) | 95 (22-6846) | 7-116 | 5-1500 | IU/L |
| ALT | 43 (16-1536) | 45 (16-1495) | 32 (10-898) | 33 (10-887) | 23-93 | 3-500 | IU/L |
| AST | 32 (15-461) | 34 (15-480) | 63 (10-343) | 58 (10-327) | 23-53 | 3-1000 | IU/L |
| TBIL | 0.2 (0.1-6.6) | 0.2 (0.1-6.7) | 0.2 (0.1-4.6) | 0.2 (0.1-4.2) | 0.1-0.4 | 0-30 | mg/dL |
| TP | 5.9 (3.8-8.8) | 6.0 (3.0-8.8) | 5.5 (3.7-7.2)^§^ | 5.5 (3.6-6.8)^§^ | 5-7.4 | 3-12 | g/dL |
| ALB | 2.8 (2.0-3.7)^*^ | 2.9 (2.0-3.8)^*^ | 2.8 (1.6-3.7)^§^ | 2.7 (1.6-3.6)^§^ | 2.6-3.9 | 1.5-6.0 | g/dL |
| CA | 9.6 (6.4-11.3) | 9.8 (6.4-11.4) | 9.4 (7.5-13.0) | 9.2 (7.2-12.5) | 8.7-10.4 | 4-18 | mg/dL |
| PHOS | 4.0 (16-65.1) | 4.1 (2.0-54.1) | 4.3 (2.0-17.3) | 4.1 (2.1-16.9) | 2.2-4.8 | 1-20 | mg/dL |
| CREA | 1.0 (0.5-24.6) | 1.0 (0.4-24.6) | 0.8 (0.2-10.0) | 0.7 (0.2-9.9) | 0.6-1.5 | 0.2-25.0 | mg/dL |
| BUN | 16 (6-482) | 16 (6-445) | 13 (4-199) | 13 (4-201) | 7-27 | 2-130 | mg/dL |
| GLU | 104 (57-176) | 97 (55-164) | 107 (57-189) | 102 (54-183) | 78-124 | 10-800 | mg/dL |
| CHOL | 246 (90-832) | 254 (93-864) | 215 (74-627) | 212 (75-622) | 102-340 | 25-700 | mg/dL |
| MG | 1.9 (1.2-5.6) | 1.9 (1.3-5.5) | 1.9 (1.3-3.5) | 1.9 (1.3-3.4) | 1.7-2.4 | 0.5-8.0 | mg/dL |
| NA | 145 (137-152)^*^ | 145 (136-151)^*^ | 147 (131-156) | 147 (132-153) | 142-151 | 5-200 | mEq/L |
| K | 4.3 (3.3-7.5) | 4.3 (3.4-8.1) | 4.2 (3.3-5.3) | 4.2 (3.4-5.3)^§^ | 3.8-5 | 1.0-10.0 | mEq/L |
| CL | 112 (67-122) | 111 (67-122) | 114 (95-121) | 114 (95-121) | 108-117 | 50-200 | mEq/L |
| HCO_3_ | 20 (9-28) | 19 (6-26) | 21 (14-25)^§^ | 20 (14-25)^§^ | 16-24 | 2-45 | mEq/L |

Abbreviations: ALB, albumin; ALP, alkaline phosphatase; ALT, alanine aminotransferase; AST, aspartate aminotransferase; AU480, Beckman Coultor AU480 biochemistry analyzer; BUN, blood urea nitrogen; CA, total calcium; CHOL, cholesterol; CL, chloride; CREA, creatinine; DV, direct venipuncture; fPIVC, freshly-placed peripheral intravenous catheter; GLU, glucose; HCO_3_, bicarbonate; hPIVC, peripheral intravenous catheter in a hospitalized patient; K, potassium; MG, total magnesium; NA, sodium; PHOS, phosphorus; RI, reference interval; TBIL, total bilirubin; TP, total protein

^*^ Means (+/- standard deviations) for normally distributed data for Arm 1 analytes: ALB_DV_ (2.9 +/- 0.5 g/dL), ALB_fPIVC_ (2.9 +/- 0.5 g/dL), NA_DV_ (146 +/- 2 mEq/L), NA_fPIVC_ (145 +/- 3 mEq/L)

^§^ Means (+/- standard deviations) for normally distributed data for Arm 2 analytes: TP_DV_ (5.5 +/- 0.7 g/dL), TP_hPIVC_ (5.4 +/- 0.7 g/dL), ALB_DV_ (2.7 +/- 0.5 g/dL), ALB_hPIVC_ (2.7 +/- 0.5 g/dL), K_hPIVC_ (4.2 +/- 0.4 mEq/L), HCO_3DV_ (21 +/- 3 mEq/L), HCO_3hPIVC_ (20 +/- 3 mEq/L).

**Supplementary Table 5.** Normality assessment for serum biochemistry data

| Analyte | Shapiro-Wilk p value | | | | | |
| --- | --- | --- | --- | --- | --- | --- |
|  | **Arm 1** | | | **Arm 2** | | |
|  | **DV** | **fPIVC** | **Pair Difference**  **(DV – fPIVC)** | **DV** | **hPIVC** | **Pair Difference**  **(DV – hPIVC)** |
| ALP | < 0.01 | < 0.01 | < 0.01 | < 0.01 | < 0.01 | < 0.01 |
| ALT | < 0.01 | < 0.01 | < 0.01 | < 0.01 | < 0.01 | < 0.01 |
| AST | < 0.01 | < 0.01 | < 0.01 | < 0.01 | < 0.01 | < 0.01 |
| TBIL | < 0.01 | < 0.01 | < 0.01 | < 0.01 | < 0.01 | < 0.01 |
| TP | 0.01 | < 0.01 | 0.02 | **0.57** | **0.26** | 0.02 |
| ALB | **0.18** | **0.23** | **0.41** | **0.62** | **0.52** | < 0.01 |
| CA | < 0.01 | < 0.01 | 0.01 | < 0.01 | < 0.01 | < 0.01 |
| PHOS | < 0.01 | < 0.01 | < 0.01 | < 0.01 | < 0.01 | **0.07** |
| CREA | < 0.01 | < 0.01 | 0.02 | < 0.01 | < 0.01 | < 0.01 |
| BUN | < 0.01 | < 0.01 | < 0.01 | < 0.01 | < 0.01 | < 0.01 |
| GLU | < 0.01 | < 0.01 | **0.50** | 0.01 | < 0.01 | < 0.01 |
| CHOL | < 0.01 | < 0.01 | < 0.01 | < 0.01 | < 0.01 | < 0.01 |
| MG | < 0.01 | < 0.01 | < 0.01 | < 0.01 | < 0.01 | < 0.01 |
| NA | **0.97** | **0.44** | **0.49** | < 0.01 | < 0.01 | **0.53** |
| K | < 0.01 | < 0.01 | 0.02 | 0.04 | **0.13** | 0.01 |
| CL | < 0.01 | < 0.01 | **0.39** | < 0.01 | < 0.01 | < 0.01 |
| HCO_3_ | < 0.01 | < 0.01 | < 0.01 | **0.08** | **0.19** | < 0.01 |

Abbreviations: ALB, albumin; ALP, alkaline phosphatase; ALT, alanine aminotransferase; AST, aspartate aminotransferase; BUN, blood urea nitrogen; CA, total calcium; CHOL, cholesterol; CL, chloride; CREA, creatinine; DV, direct venipuncture; fPIVC, freshly-placed peripheral intravenous catheter; GLU, glucose; HCO_3_, bicarbonate; hPIVC, peripheral intravenous catheter in a hospitalized patient; K, potassium; MG, total magnesium; NA, sodium; PHOS, phosphorus; TBIL, total bilirubin; TP, total protein

Normality was rejected for p < 0.05 for Shapiro-Wilk or if coefficient of skewness or coefficient of kurtosis had p ≤ 0.05. Normally-distributed data have bolded p values.

**Supplementary Table 6.** CBC data pairs outside of consensus total allowable error

| **Analyte** | **Units** | **Arm 1** | | **Arm 2** | |
| --- | --- | --- | --- | --- | --- |
|  |  | **Sample pairs outside of cTEa** | **Specific Sample Pairs**  **(DV, fPIVC)** | **Samples pairs outside of cTEa** | **Specific Sample Pairs**  **(DV, hPIVC)** |
| **WBC** | K/uL | 2 | (4.8, 4.1); (0.35, 0.52) | 2 | (0.38, 0.32); (5.8, 5.0) |
| **RBC** | M/uL | 0 | - | 13 | (6.5, 5.5); (7.4, 6.6); (5.9, 4.3); (5.2, 6.4); (6.5, 5.7); (7.3, 6.6); (6.9, 5.7); (6.2, 5.4); (4.7, 4.2); (2.8, 2.5); (5.4, 4.9); (5.0, 4.4); (6.8, 5.9) |
| **HB** | g/dL | 1 | (13.1; 14.3) | 12 | (14.8, 12.8); (17.8, 16.0); (14.1, 10.3); (12.4, 15.1); (15.2, 13.1); (16.5, 14.9); (13.1, 17.7); (18.8, 15.8); (16.2, 12.8); (11.9, 10.5); (6.8, 6.1); (12.0, 10.7); (15.7, 13.9) |
| **HCT** | % | 1 | (27.9; 30.9) | 4 | (42, 36); (50, 45); **(40, 29)^*^;** (37, 44); (44, 38); (47, 42); (51, 42); (43, 37); (33, 29); (20, 18); (35, 31); (45, 40) |
| **PCV** | % | 2 | (36, 41); (28, 31) | 9 | (42, 37); (33, 29); (50, 44); (39, 30); (44, 38); (50, 43); (45, 37); (35, 30); (44, 38) |
| **MCV** | fL | 0 | - | 0 | - |
| **MCHC** | g/dL | 0 | - | 1 | (35.4, 45.6) |
| **PLT** | K/uL | 1 | (225, 137) | 3 | (106, 86); (259; 209); (111, 90) |
| **NEUT** | K/uL | 6 | (4.7, 3.8); (8.7, 7.1); (3.9, 4.5); (8.9, 7.6); (0.1, 0.3); (5.2, 6.3) | 27 | (10.4; 8.6); (5.6, 4.8); (8.4, 6.8); (11.1, 12.0); (8.7, 7.0); (6.5, 8.3); (3.9, 4.9); (9.8, 10.6); (4.6, 4.3); (6.2, 7.8); (18.7, 16.9); (0.2, 0.1); (14.6, 11.9); (7.0, 6.0); (15.3, 14.0); (3.7, 3.3); (10.6, 9.6); (7.8, 6.8); (11.6, 12.8); (10.3, 11.6); (19.3, 21.6); (8.9, 7.6); (11.8, 12.9); (11.5, 8.0); (16.6, 18.3); (7.3, 8.0); (16.3, 18.2) |
| **LYMPH** | K/uL | 44 | (0.6, 0.4); (0.6, 0.8); (0.9, 1.6); (3.4, 4.8); (0.9, 1.4); (1.1, 0.7); (0.8, 0.5); (1.4, 1.8); (1.0, 1.4); (0.6, 0.7); (1.1, 0.8); (1.7, 2.0); (0.5, 0.6); (0.9, 1.7); (1.0, 1.2); (3.3, 4.2); (0.4, 0.7); (1.3, 1.5); (0.6, 0.9); (0.9, 1.4); (0.8, 0.4); (1.7, 2.8); (0.9, 0.4); (1.0, 0.4); (1.6, 1.9); (2.1, 0.7); (1.6, 1.2); (0.5, 0.2); (1.9, 1.2); (3.5, 2.5); (1.2, 0.4); (1.0, 1.7); (1.5, 1.2); (2.1, 2.5); **(5.7, 3.1)^*^;** (2.8, 2.2); (0.7, 0.6); (0.3, 1.3); (1.2, 0.4); (2.7, 3.2); (0.2, 0.1); (0.3, 0.7); (1.0, 0.3); (0.8, 1.4) | 41 | (1.2, 1.7); (0.5, 0.3); (1.1, 1.8); (1.7, 2.1); (1.6, 1.3); (0.9, 0.5); (1.1, 0.8); (0.8, 1.2); (0.7, 0.4); (0.5, 0.6); (2.8, 2.1); (1.0, 1.5); (1.7, 0.4); (1.9, 3.2); (0.1, 0.2); (0.9, 0.5); (3.0, 3.5); (4.0, 2.0); (1.0, 2.0); (1.0, 0.7); (1.6, 2.3); (0.6, 0.8); (1.9, 0.6); (1.4, 1.7); (1.0, 1.4); (0.4, 0.8); (1.1, 1.5); (1.9, 1.2); **(4.9, 2.8)^*^;** (0.5, 0.9); (0.7, 1.1); (1.3, 2.4); (1.1, 0.7); (1.7, 2.2); (3.6, 2.1); (2.1, 1.4); (1.9, 3.6); **(2.3, 4.3)^*^;** (3.0, 1.5); (0.3, 0.2); (1.9, 1.5) |
| **MONO** | K/uL | 18 | (0.8, 0.2); (0.3, 0.1); (0.6, 1.1); (0.3, 0.6); (0.1, 0.2); (0.1, 0.4); (2.2, 0.9); (0.5, 1.0); (0.0, 0.5); (0.7, 1.6); (0.5, 1.2); **(2.4, 4.2)^*^;** (0.4, 0.8); (2.1, 3.6); (0.3, 0.6); (0.2, 0.9); (0.4, 1.2); (0.8, 0.3) | 19 | (0.7, 1.4); (1.1, 0.6); (2.9, 1.0); (0.6, 0.0); (0.0, 1.1); (1.4, 0.8); (1.1, 2.0); **(0.8, 2.1)^*^;** (0.2, 0.5); **(0.3, 1.2)^*^;** **(1.3, 3.6)^*^;** (0.6, 1.1); (0.9, 1.5); (0.7, 0.3); (0.2, 0.6); (1.5, 0.3); (0.2, 0.5); (0.4, 0.1); (1.0, 1.8) |
| **EOS** | K/uL | 29 | (0.4, 0.1); (0.1, 0.3); (0.3, 0.6); (0.8, 1.4); (0.0, 0.1); (0.6, 0.4); (0.2, 0.4); (0.1, 0.6); (0.7, 0.1); (0.2, 0.1); (0.5, 0.2); (0.2, 0.0); (0.3, 0.1); (0.0, 0.1); (0.3, 0.1); (0.4, 0.1); (0.0, 0.2); (0.0, 0.1); (1.3, 0.4); (0.0, 0.1); (0.0, 0.2); (0.4, 0.7); (0.0; 0.5); (0.1, 0.3); (0.1, 0.3); (0.1, 0.3); (0.2, 0.6); (0.0, 0.1); (0.0, 0.1) | 33 | (0.0, 0.8); (0.3, 0.1); (0.8, 0.3); (0.1, 0.7); (0.0, 0.1); (0.0, 0.7); (0.0, 0.4); (0.2, 0.0); (0.5, 0.9); (0.4, 0.0); (0.2, 1.3); (0.2, 0.0); (0.2, 0.6); (0.9, 0.0); **(2.7, 1.0)^*^;** (0.2, 0.4); (0.4, 0.2); (0.2, 0.4); (0.2, 0.6); (0.4, 0.2); (0.9, 0.2); (0.3, 0.0); (0.0, 0.2); (0.0, 0.3); (0.1, 0.0); (0.7, 0.2); (0.1, 0.6); (0.4, 0.8); (0.0, 0.3); **(0.9, 2.5)^*^;** (0.1, 0.3); (0.2, 0.0); (0.1, 0.0) |

Abbreviations: DV, direct venipuncture; EOS, eosinophil count; fPIVC, freshly-placed peripheral intravenous catheter; HB, hemoglobin; HCT, hematocrit; hPIVC, peripheral intravenous catheter in a hospitalized patient; LYMPH, lymphocyte count; MCHC, mean corpuscular hemoglobin concentration; MCV, mean corpuscular volume; MONO, monocyte count; NEUT, neutrophil count; PLT, total platelet count; RBC, red blood cell count; WBC, leukocyte count

* Indicates that the difference between the values in this sample pair was assessed to potentially affect clinical decision making. These sample pairs are in bold.

**Supplementary Table 7.** Descriptive statistics for CBC data.

| Analyte | Arm 1 | | Arm 2 | | Reference Interval | Units |
| --- | --- | --- | --- | --- | --- | --- |
|  | DV  Median (range) | fPIVC  Median (range) | DV  Median (range) | hPIVC  Median (range) |  |  |
| WBC | 9.5 (0.4-88.9) | 9.8 (0.5-86.7) | 13.1 (0.4-56.3) | 13.0 (0.3-55.2) | 5.0-13.0 | K/uL |
| RBC | 6.5 (4.0-6.4)^*^ | 6.7 (4.0-8.4)^*^ | 5.9 (2.8-8.4)^§^ | 5.7 (2.5-8.4)^§^ | 5.7-8.3 | M/uL |
| HB | 15.2 (9.0-20.0)^*^ | 15.8 (9.4-20.2)^*^ | 13.9 (6.8-20.8)^§^ | 13.4 (6.1-21.0)^§^ | 14.0-20.0 | g/dL |
| HCT | 43 (28-55)^*^ | 46 (30-58)^*^ | 40 (20-58)^§^ | 38 (18-58)^§^ | 40-56 | % |
| PCV | 43 (28-56)^*^ | 45 (30-57)^*^ | 39 (19-59)^§^ | 38 (18-57)^§^ | 40-56 | % |
| MCV | 67 (54-75) | 68 (54-75) | 68 (53-74) | 68 (53-74) | 64-74 | fL |
| MCHC | 36 (30-39) | 35 (29-37) | 35 (30-38) | 35 (29-46) | 33-38 | g/dL |
| RDW | 12.6 (11.0-18.1) | 12.5 (10.8-17.9) | 12.3 (10.7-18.6) | 12.3 (10.7-18.7) | 11.0-14.0 | % |
| MPV | 11.8 (8.5-21.3) | 12.1 (8.7-20.8) | 11.6 (7.7-25.1) | 12.1 (7.6-24.4) | 10.0-15.0 | fL |
| PLT | 257 (33-716) | 257 (28-729) | 234 (62-472)^§^ | 227 (55-425)^§^ | 134-396 | K/uL |
| TS | 7.1 (4.4-12.2) | 7.3 (4.5-12.2) | 6.6 (4.8-8.1)^§^ | 6.5 (4.6-7.7)^§^ | 6.0-8.0 | g/dL |
| NEUT | 7.3 (0.1-69.3) | 7.3 (0.3-78.0) | 10.5 (0.2-43.3) | 9.7 (0.1-44.7) | 2.7-8.9 | K/uL |
| BAND | 0.0 (0.0-6.4) | 0.0 (0.0-7.1) | 0.0 (0.0-3.9) | 0.0 (0.0-2.9) | 0.0-0.0 | K/uL |
| LYMPH | 1.0 (0.2-5.7) | 1.2 (0.1-4.8) | 1.4 (0.1-5.5) | 1.5 (0.2-6.0) | 0.9-3.4 | K/uL |
| MONO | 0.5 (0.0-5.3) | 0.6 (0.1-4.3) | 0.7 (0.0-6.2) | 0.7 (0.0-5.5) | 0.1-0.8 | K/uL |
| EOS | 0.3 (0.0-1.4) | 0.2 (0.0-1.4) | 0.2 (0.0-2.7) | 0.3 (0.0-2.5) | 0.1-1.3 | K/uL |
| BASO | 0.0 (0.0-0.0) | 0.0 (0.0-0.1) | 0.0 (0.0-0.2) | 0.0 (0.0-0.4) | 0.0-0.1 | K/uL |
| nRBC | 0.0 (0.0-0.2) | 0.0 (0.0-0.2) | 0.0 (0.0-1.0) | 0.0 (0.0-0.2) | 0.0-0.0 | K/uL |
| HPP | 0.3 (0.0-0.9) | 0.3 (0.0-0.9) | 0.3 (0.0-0.7)^§^ | 0.3 (0.0-0.7) | 0.1-0.4 | g/dL |

Abbreviations: BAND, band neutrophil count; BASO, basophil count; DV, direct venipuncture; EOS, eosinophil count; fPIVC, freshly-placed peripheral intravenous catheter; HB, hemoglobin; HCT, hematocrit; hPIVC, peripheral intravenous catheter in a hospitalized patient; HPP, heat precipitated proteins; LYMPH, lymphocyte count; MCHC, mean corpuscular hemoglobin concentration; MCV, mean corpuscular volume; MONO, monocyte count; MPV, mean platelet volume; NEUT, neutrophil count; nRBC, nucleated red blood cell count; PLT, total platelet count; RBC, red blood cell count; RDW, red cell distribution width; TS, total solids; WBC, leukocyte count

^*^Means (+/- standard deviations) for normally distributed data for Arm 1 analytes: RBC_DV_ (6.4 +/- 1.0 M/uL); RBC_fPIVC_ (6.6 +/- 1.0 M/uL); HB_DV_ (15.2 +/- 2.6 g/dL); HB_fPIVC_ (15.6 +/- 2.6 g/dL); HCT_DV_ (43 +/- 7 %); HCT_fPIVC_ (44 +/- 7 %); PCV_DV_ (43 +/- 7 %); PCV_fPIVC_ (44 +/- 7 %).

^§^Means (+/- standard deviations) for normally distributed data for Arm 2 analytes: RBC_DV_ (5.9 +/- 1.0 M/uL); RBC_hPIVC_ (5.6 +/- 1.0 M/uL); HB_DV_ (13.9 +/- 2.6 g/dL); HB_hPIVC_ (13.4 +/- 2.6 g/dL); HCT_DV_ (40 +/- 7%); HCT_hPIVC_ (38 +/- 7 %); PCV_DV_ (39 +/- 7 %); PCV_hPIVC_ (38 +/- 7 %); PLT_DV_ (243 +/- 98 K/uL); PLT_hPIVC_ (228 +/- 94 K/uL); TS_DV_ (6.5 +/- 0.8 g/dL); TS_hPIVC_ (6.3 +/- 0.7 g/dL); HPP_DV_ (0.3 +/- 0.2 g/dL)

**Supplementary Table 8.** Normality assessment for CBC data

| Analyte | Shapiro-Wilk p value | | | | | |
| --- | --- | --- | --- | --- | --- | --- |
|  | Arm 1 | | | Arm 2 | | |
|  | DV | fPIVC | Differences  (DV – fPIVC) | DV | hPIVC | Differences  (DV – hPIVC) |
| WBC | < 0.01 | < 0.01 | < 0.01 | < 0.01 | < 0.01 | **0.89** |
| RBC | **0.12** | **0.27** | **0.57** | **0.98** | **0.64** | < 0.01 |
| HB | **0.30** | **0.26** | **0.54** | **0.98** | **0.60** | < 0.01 |
| HCT | **0.14** | **0.22** | 0.12* | **0.94** | **0.52** | < 0.01 |
| PCV | **0.20** | **0.09** | < 0.01 | **0.97** | **0.37** | < 0.01 |
| MCV | < 0.01 | < 0.01 | **0.18** | < 0.01 | < 0.01 | < 0.01 |
| MCHC | < 0.01 | < 0.01 | **0.60** | < 0.01 | < 0.01 | < 0.01 |
| RDW | < 0.01 | < 0.01 | < 0.01 | < 0.01 | < 0.01 | < 0.01 |
| MPV | < 0.01 | < 0.01 | < 0.01 | < 0.01 | < 0.01 | < 0.01 |
| PLT | < 0.01 | < 0.01 | < 0.01 | **0.71** | **0.61** | < 0.01 |
| TS | < 0.01 | < 0.01 | **0.14** | **0.79** | **0.32** | < 0.01 |
| NEUT | < 0.01 | < 0.01 | < 0.01 | < 0.01 | < 0.01 | **0.78** |
| BAND | < 0.01 | < 0.01 | < 0.01 | < 0.01 | < 0.01 | < 0.01 |
| LYMPH | < 0.01 | < 0.01 | < 0.01 | < 0.01 | < 0.01 | **0.05** |
| MONO | < 0.01 | < 0.01 | < 0.01 | < 0.01 | < 0.01 | < 0.01 |
| EOS | < 0.01 | < 0.01 | 0.07* | < 0.01 | < 0.01 | < 0.01 |
| HPP | < 0.01 | < 0.01 | < 0.01 | **0.06** | 0.04 | < 0.01 |

Abbreviations: BAND, band neutrophil count; DV, direct venipuncture; EOS, eosinophil count; fPIVC, freshly-placed peripheral intravenous catheter; HB, hemoglobin; HCT, hematocrit; hPIVC, peripheral intravenous catheter in a hospitalized patient; HPP, heat precipitated proteins; LYMPH, lymphocyte count; MCHC, mean corpuscular hemoglobin concentration; MCV, mean corpuscular volume; MONO, monocyte count; MPV, mean platelet volume; NEUT, neutrophil count; PLT, total platelet count; RBC, red blood cell count; RDW, red cell distribution width; TS, total solids; WBC, leukocyte count

Normality was rejected for p < 0.05 for Shapiro-Wilk or if coefficient of skewness or coefficient of kurtosis had p ≤ 0.05. Normally-distributed data have bolded p values.

*Normality rejected due to coefficient of kurtosis p ≤ 0.05
